# Incidental Finding of MEGDEL Syndrome at a Tertiary Care Center in Saudi Arabia

**DOI:** 10.7759/cureus.55308

**Published:** 2024-03-01

**Authors:** Aisha T Alfaraidi, Nahed k ALSulimani, Wallaa Garout

**Affiliations:** 1 College of Medicine, King Abdulaziz University Faculty of Medicine, Jeddah, SAU; 2 Pediatric Department, King Abdulaziz University Hospital, Jeddah, SAU

**Keywords:** hypotonia, mitochondrial, glutaconic acid, leigh syndrome, serac1, megdel syndrome

## Abstract

MEGDEL syndrome, a rare autosomal recessive disorder characterized by 3-methylglutaconic aciduria, deafness, encephalopathy, and Leigh-like syndrome, results from mutations in the SERAC1 gene. This case report explores the clinical presentation, diagnostic challenges, and genetic findings of an 11-year-old boy with MEGDEL syndrome at a tertiary care center in Saudi Arabia.

The patient, born to consanguineous parents, presented with developmental delay, cerebral palsy, intellectual disability, and seizures. Diagnostic evaluation at 15 months revealed 3-methylglutaconic aciduria, and subsequent genetic testing through whole exome sequencing confirmed a rare homozygous deletion variant in the SERAC1 gene. The patient exhibited brain atrophy, tracheal stenosis, laryngomalacia, and skeletal abnormalities. The complexity of MEGDEL syndrome manifestations and the challenge of distinguishing it from other metabolic disorders are discussed, emphasizing the significance of genetic testing in confirming the diagnosis.

This case underscores the occurrence of MEGDEL syndrome in a child with cerebral palsy, highlighting the importance of a multidisciplinary approach for diagnosis and the need for genetic counseling in consanguineous families. Although the management remains primarily supportive, the report calls for more comprehensive epidemiological studies to determine the prevalence and incidence of MEGDEL syndrome. The findings contribute to the growing understanding of this rare disorder, thus emphasizing the necessity for ongoing research to enhance diagnostic accuracy and management strategies.

## Introduction

MEGDEL syndrome (3-methylglutaconic aciduria (MEG), Deafness (D), Encephalopathy (E), Leigh-like syndrome (L)) is an autosomal recessive disorder [1]. It is characterized by psychomotor delay, muscular hypotonia, sensorineural deafness, and Leigh-like syndrome lesions on brain magnetic resonance imaging (MRI) [1]. This syndrome is caused by a mutation in the Serine active site-containing protein 1 (SERAC1) gene [2]. SERAC1 is essential for phosphatidylglycerol remodeling, which is found in the mitochondrion-associated membrane fraction that is required for mitochondrial function and intracellular cholesterol trafficking [2]. MEGDEL syndrome exhibits more variable phenotypic characteristics. Patients with juvenile-onset spasticity and moderate cognitive impairment may have a milder presentation, resulting in a broader phenotypic spectrum of the disease. The condition was found to affect many body parts, including the ears, eyes, brain, endocrine organs, heart, peripheral nerves, skeletal muscle, and gastrointestinal tract [1]. The diagnosis of MEGDEL syndrome is confirmed by a multidisciplinary approach, which is confirmed by genetic testing for the SERAC1 mutation [1]. MEGDEL syndrome is managed as a supportive condition. However, the prognosis is typically poor, with early death [3]. There were insufficient epidemiological studies on the prevalence and incidence of MEGDEL syndrome. Therefore, the incidence of the disease remains unknown. In this case report, we present the clinical presentation of a child with MEGDEL syndrome. The child presented with early developmental delay, intellectual disability, and seizures. Physical examination showed hypotonia and anorexia. Magnetic resonance imaging (MRI) of the brain showed brain atrophy.

## Case presentation

An 11-year-old boy born to first-degree consanguineous parents was evaluated at the King Abdulaziz University Hospital (KAUH) as a case of developmental delay, cerebral palsy, and intellectual disability. By clinical assessment and taking history of the patient's growth and communication parameters, his developmental age was determined to be 2 months old. He was bedridden, with few distal movements. As a result, the patient was unable to attend kindergarten or school. At the age of 15 months, he was evaluated by the pediatric metabolic team to rule out any metabolic conditions. An initial biochemical analysis using gas chromatography-mass spectrometry (GC-MS) revealed 3-Methylglucatonic aciduria (3-MGA-uria), with a concentration of 1400 mmol/mol creatinine along with mild elevation in lactic acid. The patient was born full-term via spontaneous vaginal delivery with a birth weight of 2.7 kg and had an uneventful neonatal course. He experienced an early developmental delay, with delayed milestones. Parents reported that their child had spastic and dystonic movements; these were assumed to be seizures and an anti-epileptic medication was prescribed. At the age of 11 years, the patient was admitted to the emergency department as a case of febrile seizure due to severe acute respiratory syndrome coronavirus 2 (SARS-Cov-2) bronchopneumonia. Upon examination, the patient appeared hypotonic and anorexic. He had wheezing and stridor caused by tracheal stenosis and laryngomalacia, as well as difficulty clearing sputum. The patient had underdeveloped lungs and frequently vomited. During his hospital stay, the patient received tegretol, levetiracetam, clonazepam, and omeprazole at a dose of 1 mg/kg/day twice daily. In addition to budesonide, the patient was given salbutamol via a nebulizer, and cough syrup three times daily. Due to poor oral intake, it was recommended to give a multi-fiber drink called "NutriniDrink" regularly.

**Figure 1:**
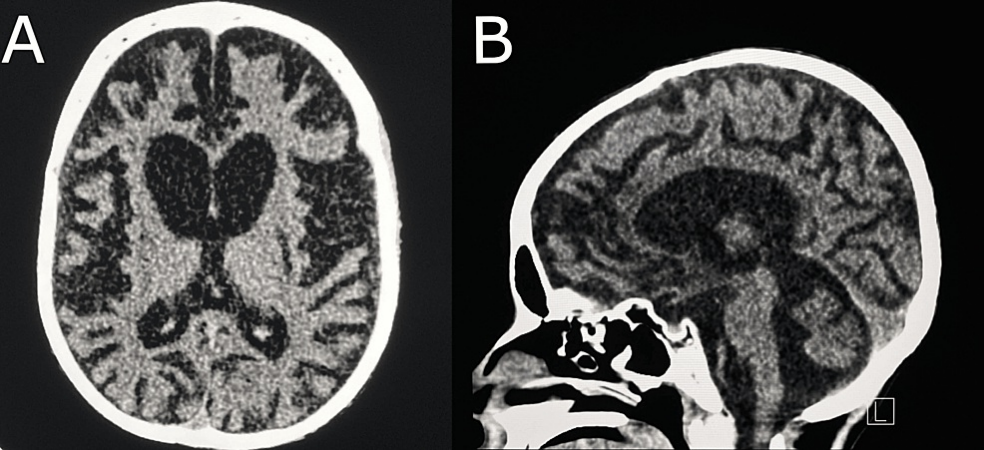
Axial plain brain CT image showing bilateral diffuse brain parenchymal volume loss, disproportionate to the patient's age (A), which resulted in dilatation of the ventricles and cortical sulci (B).

**Figure 2:**
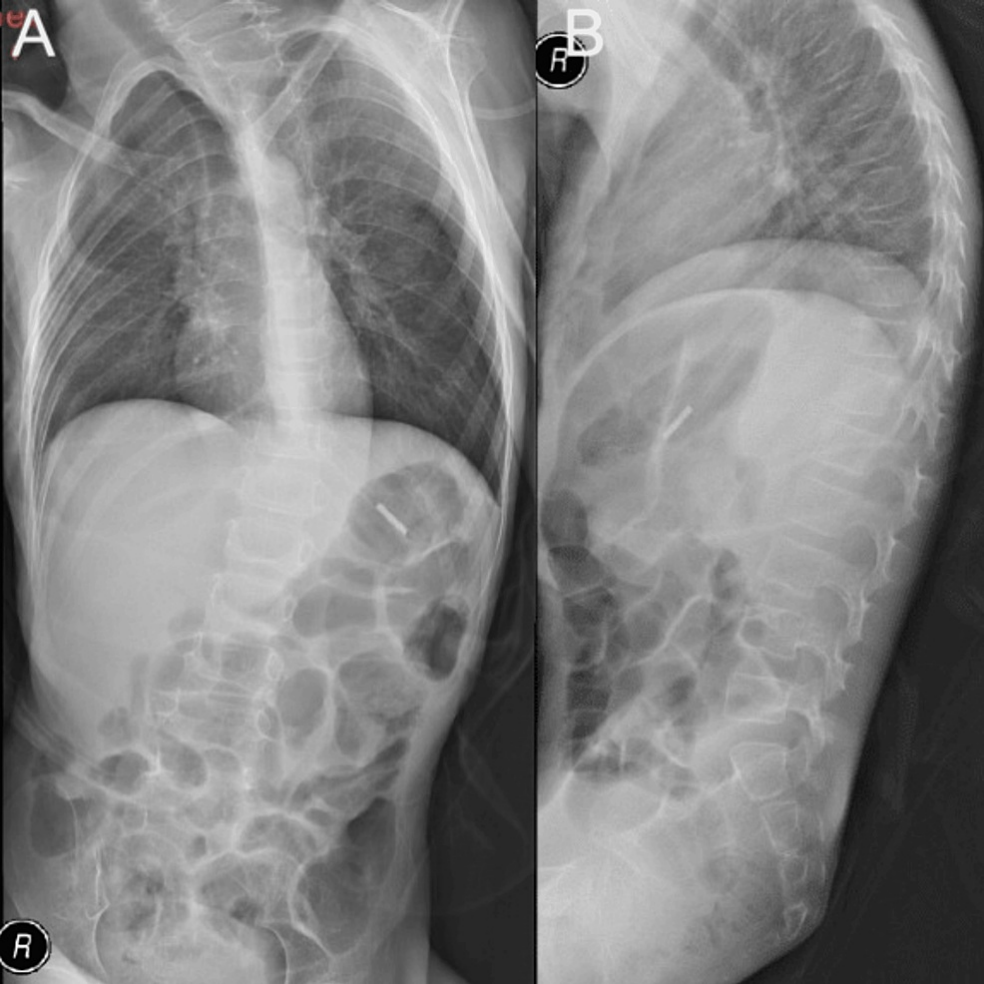
Supine AP X-ray (A) showing left-sided scoliosis with diffuse reduced bone density, and lateral X-ray from the side (B)

A brain computerized tomography (CT) scan revealed significant brain atrophy and abnormal signal intensity bilaterally in the lentiform nuclei. There was no evidence of abnormal parenchymal or meningeal enhancement, or any space-occupying lesions (Figures 1A, 1B). The patient had left-sided scoliosis and diffusely reduced bone density (Figures 2A, 2B).

Whole Exome Sequencing (WES) confirmed a rare pathogenic homozygous deletion variant in the SERAC1 gene. The significance of this variant remains unknown. WES confirmed a frameshift variation present in exon six, which was present in the middle of the “Armadillo-like helical (IPR011989)” domain. Based on the clinical history and genetic findings, a diagnosis of MEGDEL syndrome was confirmed.

## Discussion

Previous reports describing the same symptoms and findings suggested a metabolic disorder as a confirmed diagnosis. As a result, physicians should conduct metabolic investigations and targeted genetic testing. MEGDEL syndrome patients present with severe motor symptoms (e.g., dystonia and spasticity) as well as intellectual disability with early onset deafness. Giron et al reported late-onset MEGDEL syndrome in a 31-year-old male with mild psychomotor delay. The patient presented with generalized dystonia and progressive lower limb spasticity triggered by fever. The SERAC1 mutation was used to identify the mitochondrial disease [4]. Earlier studies discovered mutations in the SERAC1 protein's Armadillo-like helical domain, serine-lipase domain, and transmembrane domain, demonstrating the significance of each domain's role in protein stability [5].

In this case report, we present a patient with MEGDEL syndrome, which is defined by 3-methylglutaconic aciduria, deafness, encephalopathy, and Leigh-like lesions with an autosomal recessive pattern. Mutations in the SERAC1 gene cause a group of autosomal recessive metabolic disorders that frequently affect children of consanguineous parents. MEGDEL syndrome was difficult to diagnose because the brain imaging lesions looked similar to those seen in Leigh disease.

In 2013, a study led by Frederic Tort discovered mutations in SERAC1 as the genetic origin of MEGDEL syndrome in 15 cases. Furthermore, their patients acquired microcephaly and optic atrophy, both of which had not previously been recorded in MEGDEL syndrome. They emphasized the use of exome sequencing in determining the genetic basis of human uncommon diseases [6]. In another case reported by Wortmann et al in 2012, the genetic basis of MEGDEL syndrome was discovered when four newborns presented with hypoglycemia, sensorineural deafness, recurrent lactic acidemia, and neonatal infections. Biochemical tests and muscle biopsies confirmed the presence of an oxidative phosphorylation deficiency [7]. In 2017, Maas et al. conducted a multicenter retrospective study on 67 cases of progressive deafness and dystonia, identifying 41 different SERAC1 variants [8].

In summary, the results of the current study revealed a novel mutation in the SERAC1 gene in the affected lineage. It is hypothesized that this mutation alters the protein structure, resulting in protein dysfunction. This dysfunction may cause abnormal cholesterol trafficking within cells, where low-density lipoprotein (LDL) binds to the LDL receptor on the cell membrane and is internalized via endocytosis. Additional research could provide new insights into how different pathogenic variants relate to MEGDEL syndrome.

## Conclusions

This case report describes the occurrence of MEGDEL syndrome in an 11-year-old patient with cerebral palsy. The management of MEGDEL syndrome is primarily supportive; however, the definitive treatment is unknown. More research on patients with MEGDEL syndrome is needed to better understand the disease's various phenotypes and management. Genetic counseling regarding future children should be provided to consanguineous families with a history of MEGDEL syndrome.
